# Supplementary material for: Parent-Reported Changes in Ontario Children’s Physical Activity Levels during the COVID-19 Pandemic
Source: Children (Basel). 2023 Jan 26;10(2):221. doi: 10.3390/children10020221 (PMC9954678; doi:10.3390/children10020221)
Supplement: Supplementary file 1 [file children-10-00221-s001.zip › children-2135034-supplementary.pdf]

Western UNIVERSITY · CANADA

Child Health & PHYSICAL ACTIVITY LABORATORY

Supplementary File S1. Items used for the present study from the *Return to Play* study surveys.

Baseline Survey Items

Q5.1 Please answer the following questions to provide some information about yourself.

Q5.2 What gender do you identify with? (Refers to **current gender** which may be different from sex assigned at birth and may be different from what is indicated on legal documents.)

- ◯ Male (1)
- ◯ Female (2)
- ◯ Prefer not to say (3)
- ◯ Prefer to self-describe: (4) ____

Q5.3 What is **your age** (in years)?

____

Q5.4 What is your postal code? (e.g., 1A1 A1A)?

____

Q5.5 Which of the following best describes the area you live?

- ◯ Rural (1)
- ◯ Suburban (2)
- ◯ Urban (3)

Q5.6 What is your racial background/ethnicity?

- Caucasian (1)
- African Canadian (2)
- South Asian (3)
- East Asian (4)
- Middle Eastern (5)
- First Nations/Aboriginal (6)
- Latin American (7)
- Other: (8) ____
- Prefer not to answer (9)

Q5.7 What is your current employment status?

- Full-time (1)
- Part-time (2)
- Occasional/Support (3)
- Unemployed (4)
- Prefer not to answer (5)

Q5.8 What is your family situation?

- ◯ Single-parent (1)
- ◯ Double-parent (2)
- ◯ Guardian-led (3)
- ◯ Other: (4) ____
- ◯ Prefer not to answer (5)

Q5.9 What is your highest level of education?

- ◯ High school (1)
- ◯ College (2)
- ◯ University (3)
- ◯ Graduate school (4)
- ◯ Prefer not to answer (5)

Q5.10 In what housing type do you live (during the COVID-19 pandemic)?

- ◯ Apartment (1)
- ◯ Condominium (2)
- ◯ Townhouse (3)
- ◯ Semi-detached house (4)
- ◯ Detached house (5)
- ◯ Other housing? Please describe: (6) ____

Q5.11 Do you have a dog?

- Yes (1)
- No (2)

Q5.12 What is your approximate **yearly total household income (before taxes)**?

- Less than $20,000 (1)
- $20,000 - $39,000 (2)
- $40,000 - $ 59,000 (3)
- $60,000 - $79,000 (4)
- $80,000 - $99,000 (5)
- $100,000 - $119,000 (6)
- $120,000 - $139,000 (7)
- More than $140,000 (8)
- Prefer not to answer (9)

Q5.13 Ontario is re-opening public spaces in three stages. What phase of re-opening are you **currently** in?

- Phase 1 re-opening (1)
- Phase 2 re-opening (2)
- Phase 3 re-opening (3)

Q5.14 How many children aged 0-12 years do you currently provide care for?
Click to write Choice 1 (1)

▼ 1 (1) ... 10 (10)

Display This Question:

If How many children aged 0-12 years do you currently provide care for? = 1

Q5.15 What is the current age (0-12 years) and sex at birth of your child?

	Male (1)	Female (2)
Age of child: (1)	☐	☐

Display This Question:

If How many children aged 0-12 years do you currently provide care for? = 2

Q5.16 What is the current age (0-12 years) and sex at birth of your children? Please complete the fields below in order from **youngest to oldest.**

	Male (1)	Female (2)
Child 1. Age of child: (1)	☐	☐
Child 2. Age of child: (2)	☐	☐

Display This Question:

If How many children aged 0-12 years do you currently provide care for? = 3

Q5.17 What is the current age (0-12 years) and sex at birth of your children? Please complete fields below in order from youngest to oldest.

	Male (1)	Female (2)
Child 1. Age of child: (3)	☐	☐
Child 2. Age of child: (4)	☐	☐
Child 3. Age of child: (5)	☐	☐

Display This Question:

If How many children aged 0-12 years do you currently provide care for? = 4

Q5.18 What is the current age (0-12 years) and sex at birth of your children? Please complete fields below in order from youngest to oldest.

	Male (1)	Female (2)
Child 1. Age of child: (1)	☐	☐
Child 2. Age of child: (2)	☐	☐
Child 3. Age of child: (3)	☐	☐
Child 4. Age of child: (4)	☐	☐

Display This Question:

If How many children aged 0-12 years do you currently provide care for? = 5

Q5.19 What is the current age (0-12 years) and sex at birth of your children? Please complete fields below in order from youngest to oldest.

	Male (1)	Female (2)
Child 1. Age of child: (1)	☐	☐
Child 2. Age of child: (2)	☐	☐
Child 3. Age of child: (3)	☐	☐
Child 4. Age of child: (4)	☐	☐
Child 5. Age of child: (5)	☐	☐

Display This Question:

If How many children aged 0-12 years do you currently provide care for? = 6

Q5.20 What is the current age (0-12 years) and sex at birth of your children? Please complete fields below in order from youngest to oldest.

	Male (1)	Female (2)
Child 1. Age of child: (1)	☐	☐
Child 2. Age of child: (2)	☐	☐
Child 3. Age of child: (3)	☐	☐
Child 4. Age of child: (4)	☐	☐
Child 5. Age of child: (5)	☐	☐
Child 6. Age of child: (6)	☐	☐

Display This Question:

If How many children aged 0-12 years do you currently provide care for? = 7

Western UNIVERSITY · CANADA

Child Health & PHYSICAL ACTIVITY LABORATORY

Q5.21 What is the current age (0-12 years) and sex at birth of your children? Please complete fields below in order from youngest to oldest.

	Male (1)	Female (2)
Child 1. Age of child: (1)	▢	▢
Child 2. Age of child: (2)	▢	▢
Child 3. Age of child: (3)	▢	▢
Child 4. Age of child: (4)	▢	▢
Child 5. Age of child: (5)	▢	▢
Child 6. Age of child: (6)	▢	▢
Child 7. Age of child: (7)	▢	▢

Display This Question:

If How many children aged 0-12 years do you currently provide care for? = 8

Q5.22 What is the current age (0-12 years) and sex at birth of your children? Please complete fields below in order from youngest to oldest.

	Male (1)	Female (2)
Child 1. Age of child: (1)	☐	☐
Child 2. Age of child: (2)	☐	☐
Child 3. Age of child: (3)	☐	☐
Child 4. Age of child: (4)	☐	☐
Child 5. Age of child: (5)	☐	☐
Child 6. Age of child: (6)	☐	☐
Child 7. Age of child: (7)	☐	☐
Child 8. Age of child: (8)	☐	☐

Display This Question:

If How many children aged 0-12 years do you currently provide care for? = 9

Q5.23 What is the current age (0-12 years) and sex at birth of your children? Please complete fields below in order from youngest to oldest.

	Male (1)	Female (2)
Child 1. Age of child: (1)	☐	☐
Child 2. Age of child: (2)	☐	☐
Child 3. Age of child: (3)	☐	☐
Child 4. Age of child: (4)	☐	☐
Child 5. Age of child: (5)	☐	☐
Child 6. Age of child: (6)	☐	☐
Child 7. Age of child: (7)	☐	☐
Child 8. Age of child: (8)	☐	☐
Child 9. Age of child: (9)	☐	☐

Display This Question:

If How many children aged 0-12 years do you currently provide care for? = 10

Q5.24 What is the current age (0-12 years) and sex at birth of your children? Please complete fields below in order from youngest to oldest.

	Male (1)	Female (2)
Child 1. Age of child: (1)	☐	☐
Child 2. Age of child: (2)	☐	☐
Child 3. Age of child: (3)	☐	☐
Child 4. Age of child: (4)	☐	☐
Child 5. Age of child: (5)	☐	☐
Child 6. Age of child: (6)	☐	☐
Child 7. Age of child: (7)	☐	☐
Child 8. Age of child: (8)	☐	☐
Child 9. Age of child: (9)	☐	☐
Child 10. Age of child: (10)	☐	☐

Q5.25 Does your child have a diagnosed disability or chronic condition?

◯ Yes (1)

◯ No (2)

Skip To: Q5.35 If Does your child have a diagnosed disability or chronic condition? = Yes

Skip To: End of Block If Does your child have a diagnosed disability or chronic condition? = No

Display This Question:

If How many children aged 0-12 years do you currently provide care for? = 2

Q5.26 Have any of your children been diagnosed with a disability or chronic condition? Please provide a response for each child. Please use the same order of children (youngest to oldest) as you have done in previous sections of the survey.

	Yes (1)	No (2)
Child 1 (1)	☐	☐
Child 2 (2)	☐	☐

Skip To: End of Block If Have any of your children been diagnosed with a disability or chronic condition? Please provide a... [No] (Count) = 2

Display This Question:

If How many children aged 0-12 years do you currently provide care for? = 3

Q5.27 Have any of your children been diagnosed with a disability or chronic condition? Please provide a response for each child. Please use the same order of children (youngest to oldest) as you have done in previous sections of the survey.

	Yes (1)	No (2)
Child 1 (1)	☐	☐
Child 2 (2)	☐	☐
Child 3 (3)	☐	☐

Skip To: End of Block If Have any of your children been diagnosed with a disability or chronic condition? Please provide a... [No] (Count) = 3

Display This Question:

If How many children aged 0-12 years do you currently provide care for? = 4

Q5.28 Have any of your children been diagnosed with a disability or chronic condition? Please provide a response for each child. Please use the same order of children (youngest to oldest) as you have done in previous sections of the survey.

	Yes (1)	No (2)
Child 1 (1)	☐	☐
Child 2 (2)	☐	☐
Child 3 (3)	☐	☐
Child 4 (4)	☐	☐

Skip To: End of Block If Have any of your children been diagnosed with a disability or chronic condition? Please provide a... [No] (Count) = 4

Display This Question:

If How many children aged 0-12 years do you currently provide care for? = 5

Q5.29 Have any of your children been diagnosed with a disability or chronic condition? Please provide a response for each child. Please use the same order of children (youngest to oldest) as you have done in previous sections of the survey.

	Yes (1)	No (2)
Child 1 (1)	☐	☐
Child 2 (2)	☐	☐
Child 3 (3)	☐	☐
Child 4 (4)	☐	☐
Child 5 (5)	☐	☐

Skip To: End of Block If Have any of your children been diagnosed with a disability or chronic condition? Please provide a... [No] (Count) = 5

Display This Question:

If How many children aged 0-12 years do you currently provide care for? = 6

Q5.30 Have any of your children been diagnosed with a disability or chronic condition? Please provide a response for each child. Please use the same order of children (youngest to oldest) as you have done in previous sections of the survey.

	Yes (1)	No (2)
Child 1 (1)	◯	◯
Child 2 (2)	◯	◯
Child 3 (3)	◯	◯
Child 4 (4)	◯	◯
Child 5 (5)	◯	◯
Child 6 (6)	◯	◯

Skip To: End of Block If Have any of your children been diagnosed with a disability or chronic condition? Please provide a... [No] (Count) = 6

Display This Question:

If How many children aged 0-12 years do you currently provide care for? = 7

Q5.31 Have any of your children been diagnosed with a disability or chronic condition? Please provide a response for each child. Please use the same order of children (youngest to oldest) as you have done in previous sections of the survey.

	Yes (1)	No (2)
Child 1 (1)	☐	☐
Child 2 (2)	☐	☐
Child 3 (3)	☐	☐
Child 4 (4)	☐	☐
Child 5 (5)	☐	☐
Child 6 (6)	☐	☐
Child 7 (7)	☐	☐

Skip To: End of Block If Have any of your children been diagnosed with a disability or chronic condition? Please provide a... [No] (Count) = 7

Display This Question:

If How many children aged 0-12 years do you currently provide care for? = 8

Q5.32 Have any of your children been diagnosed with a disability or chronic condition? Please provide a response for each child. Please use the same order of children (youngest to oldest) as you have done in previous sections of the survey.

	Yes (1)	No (2)
Child 1 (1)	☐	☐
Child 2 (2)	☐	☐
Child 3 (3)	☐	☐
Child 4 (4)	☐	☐
Child 5 (5)	☐	☐
Child 6 (6)	☐	☐
Child 7 (7)	☐	☐
Child 8 (8)	☐	☐

Skip To: End of Block If Have any of your children been diagnosed with a disability or chronic condition? Please provide a... [No] (Count) = 8

Display This Question:

If How many children aged 0-12 years do you currently provide care for? = 9

Q5.33 Have any of your children been diagnosed with a disability or chronic condition? Please provide a response for each child. Please use the same order of children (youngest to oldest) as you have done in previous sections of the survey.

	Yes (1)	No (2)
Child 1 (1)	☐	☐
Child 2 (2)	☐	☐
Child 3 (3)	☐	☐
Child 4 (4)	☐	☐
Child 5 (5)	☐	☐
Child 6 (6)	☐	☐
Child 7 (7)	☐	☐
Child 8 (8)	☐	☐
Child 9 (9)	☐	☐

Skip To: End of Block If Have any of your children been diagnosed with a disability or chronic condition? Please provide a... [No] (Count) = 9

Display This Question:

If How many children aged 0-12 years do you currently provide care for? = 10

Q5.34 Have any of your children been diagnosed with a disability or chronic condition? Please provide a response for each child. Please use the same order of children (youngest to oldest) as you have done in previous sections of the survey.

	Yes (1)	No (2)
Child 1 (1)	☐	☐
Child 2 (2)	☐	☐
Child 3 (3)	☐	☐
Child 4 (4)	☐	☐
Child 5 (5)	☐	☐
Child 6 (6)	☐	☐
Child 7 (7)	☐	☐
Child 8 (8)	☐	☐
Child 9 (9)	☐	☐
Child 10 (10)	☐	☐

Skip To: End of Block If Have any of your children been diagnosed with a disability or chronic condition? Please provide a... [No] (Count) = 10

Display This Question:

If How many children aged 0-12 years do you currently provide care for? = 1

Q6.24 In your opinion, how **<u>many minutes per day</u>** did your child spend **<u>engaged in physical activity prior to the COVID-19 pandemic</u>**?

- Less than 30 minutes per day (1)
- 30-59 minutes per day (2)
- 60-149 minutes per day (3)
- 150 minutes or more per day (4)

Display This Question:

If How many children aged 0-12 years do you currently provide care for? = 2

Q6.25 In your opinion, how **<u>many minutes per day</u>** did your child(ren) spend **<u>engaged in physical activity prior to the COVID-19 pandemic</u>**?

	Child 1 (1)	Child 2 (2)
Less than 30 minutes per day (1)	☐	☐
30-59 minutes per day (2)	☐	☐
60-149 minutes per day (3)	☐	☐
150 minutes or more per day (4)	☐	☐

Display This Question:

If How many children aged 0-12 years do you currently provide care for? = 3

Q6.26 In your opinion, how **many minutes per day** did your child(ren) spend **engaged in physical activity prior to the COVID-19 pandemic**?

	Child 1 (1)	Child 2 (2)	Child 3 (3)
Less than 30 minutes per day (1)	☐	☐	☐
30-59 minutes per day (2)	☐	☐	☐
60-149 minutes per day (3)	☐	☐	☐
150 minutes or more per day (4)	☐	☐	☐

Display This Question:

If How many children aged 0-12 years do you currently provide care for? = 4

Q6.27 In your opinion, how **many minutes per day** did your child(ren) spend **engaged in physical activity prior to the COVID-19 pandemic**?

	Child 1 (1)	Child 2 (2)	Child 3 (3)	Child 4 (4)
Less than 30 minutes per day (1)	☐	☐	☐	☐
30-59 minutes per day (2)	☐	☐	☐	☐
60-149 minutes per day (3)	☐	☐	☐	☐
150 minutes or more per day (4)	☐	☐	☐	☐

Display This Question:

If How many children aged 0-12 years do you currently provide care for? = 5

Q6.28 In your opinion, how **many minutes per day** did your child(ren) spend **engaged in physical activity prior to the COVID-19 pandemic**?

	Child 1 (1)	Child 2 (2)	Child 3 (3)	Child 4 (4)	Child 5 (5)
Less than 30 minutes per day (1)	☐	☐	☐	☐	☐
30-59 minutes per day (2)	☐	☐	☐	☐	☐
60-149 minutes per day (3)	☐	☐	☐	☐	☐
150 minutes or more per day (4)	☐	☐	☐	☐	☐

Display This Question:

If How many children aged 0-12 years do you currently provide care for? = 6

Q6.29 In your opinion, how **many minutes per day** did your child(ren) spend **engaged in physical activity prior to the COVID-19 pandemic**?

	Child 1 (1)	Child 2 (2)	Child 3 (3)	Child 4 (4)	Child 5 (5)	Child 6 (6)
Less than 30 minutes per day (1)	☐	☐	☐	☐	☐	☐
30-59 minutes per day (2)	☐	☐	☐	☐	☐	☐
60-149 minutes per day (3)	☐	☐	☐	☐	☐	☐
150 minutes or more per day (4)	☐	☐	☐	☐	☐	☐

Display This Question:

If How many children aged 0-12 years do you currently provide care for? = 7

Q6.30 In your opinion, how **many minutes per day** did your child(ren) spend **engaged in physical activity prior to the COVID-19 pandemic**?

	Child 1 (1)	Child 2 (2)	Child 3 (3)	Child 4 (4)	Child 5 (5)	Child 6 (6)	Child 7 (7)
Less than 30 minutes per day (1)	☐	☐	☐	☐	☐	☐	☐
30-59 minutes per day (2)	☐	☐	☐	☐	☐	☐	☐
60-149 minutes per day (3)	☐	☐	☐	☐	☐	☐	☐
150 minutes or more per day (4)	☐	☐	☐	☐	☐	☐	☐

Display This Question:

If How many children aged 0-12 years do you currently provide care for? = 8

Q6.31 In your opinion, how **many minutes per day** did your child(ren) spend **engaged in physical activity prior to the COVID-19 pandemic**?

	Child 1 (1)	Child 2 (2)	Child 3 (3)	Child 4 (4)	Child 5 (5)	Child 6 (6)	Child 7 (7)	Child 8 (8)
Less than 30 minutes per day (1)	☐	☐	☐	☐	☐	☐	☐	☐
30-59 minutes per day (2)	☐	☐	☐	☐	☐	☐	☐	☐
60-149 minutes per day (3)	☐	☐	☐	☐	☐	☐	☐	☐
150 minutes or more per day (4)	☐	☐	☐	☐	☐	☐	☐	☐

Display This Question:

If How many children aged 0-12 years do you currently provide care for? = 9

Q6.32 In your opinion, how **many minutes per day** did your child(ren) spend **engaged in physical activity prior to the COVID-19 pandemic**?

	Child 1 (1)	Child 2 (2)	Child 3 (3)	Child 4 (4)	Child 5 (5)	Child 6 (6)	Child 7 (7)	Child 8 (8)	Child 9 (9)
Less than 30 minutes per day (1)	☐	☐	☐	☐	☐	☐	☐	☐	☐
30-59 minutes per day (2)	☐	☐	☐	☐	☐	☐	☐	☐	☐
60-149 minutes per day (3)	☐	☐	☐	☐	☐	☐	☐	☐	☐
150 minutes or more per day (4)	☐	☐	☐	☐	☐	☐	☐	☐	☐

Display This Question:

If How many children aged 0-12 years do you currently provide care for? = 10

Q6.33 In your opinion, how **<u>many minutes per day</u>** did your child(ren) spend **<u>engaged in physical activity prior to the COVID-19 pandemic</u>**?

	Child 1 (1)	Child 2 (2)	Child 3 (3)	Child 4 (4)	Child 5 (5)	Child 6 (6)	Child 7 (7)	Child 8 (8)	Child 9 (9)	Child 10 (10)
Less than 30 minutes per day (1)	▢	▢	▢	▢	▢	▢	▢	▢	▢	▢
30-59 minutes per day (2)	▢	▢	▢	▢	▢	▢	▢	▢	▢	▢
60-149 minutes per day (3)	▢	▢	▢	▢	▢	▢	▢	▢	▢	▢
150 minutes or more per day (4)	▢	▢	▢	▢	▢	▢	▢	▢	▢	▢

Display This Question:

If How many children aged 0-13 years do you currently provide care for? = 1

Q5.10 In your opinion, **how many minutes per day** did your child spend engaged in physical activity during Ontario's **strictest COVID-19 related lockdowns** (when sport and neighbourhood closures were in effect; March 2020- June 2020; January 2021 - May 2021)?

- Less than 30 minutes per day (1)
- 30-59 minutes per day (2)
- 60-149 minutes per day (3)
- 150 minutes or more per day (4)

Display This Question:

If How many children aged 0-13 years do you currently provide care for? = 1

Q5.11 In your opinion, how many **minutes per day** is your child engaging in physical activity **currently (i.e., at this moment in time)?**

- Less than 30 minutes per day (1)
- 30-59 minutes per day (2)
- 60-149 minutes per day (3)
- 150 minutes or more per day (4)

Display This Question:

If How many children aged 0-13 years do you currently provide care for? = 2

Q5.12 In your opinion, **how many minutes per day** did your children spend engaged in physical activity during Ontario's **strictest COVID-19 related lockdowns** (when sport and neighbourhood

closures were in effect; March 2020- June 2020; January 2021 - May 2021)? **Please respond as you have in previous questions of the survey, from YOUNGEST to OLDEST.**

	Less than 30 minutes per day (1)	30-59 minutes per day (2)	60-149 minutes per day (4)	150 minutes or more per day (5)
Child 1 (1)	○	○	○	○
Child 2 (2)	○	○	○	○

Display This Question:

If How many children aged 0-13 years do you currently provide care for? = 2

Q5.13 In your opinion, how many **minutes per day** are your children engaging in physical activity **currently (i.e., at this moment in time)? Please respond as you have in previous questions of the survey, from YOUNGEST to OLDEST.**

	Less than 30 minutes per day (1)	30-59 minutes per day (2)	60-149 minutes per day (4)	150 minutes or more per day (6)
Child 1 (1)	○	○	○	○
Child 2 (2)	○	○	○	○

Display This Question:

If How many children aged 0-13 years do you currently provide care for? = 3

Q5.14 In your opinion, **how many minutes per day** did your children spend engaged in physical activity during Ontario's **strictest COVID-19 related lockdowns** (when sport and neighbourhood

closures were in effect; March 2020- June 2020; January 2021 - May 2021)? **Please respond as you have in previous questions of the survey, from YOUNGEST to OLDEST.**

	Less than 30 minutes per day (1)	30-59 minutes per day (2)	60-149 minutes per day (4)	150 minutes or more per day (5)
Child 1 (1)	o	o	o	o
Child 2 (6)	o	o	o	o
Child 3 (7)	o	o	o	o

Display This Question:

If How many children aged 0-13 years do you currently provide care for? = 3

Q5.15 In your opinion, how many **minutes per day** are your children engaging in physical activity **currently (i.e., at this moment in time)? Please respond as you have in previous questions of the survey, from YOUNGEST to OLDEST.**

	Less than 30 minutes per day (1)	30-59 minutes per day (2)	60-149 minutes per day (4)	150 minutes or more per day (5)
Child 1 (1)	o	o	o	o
Child 2 (2)	o	o	o	o
Child 3 (3)	o	o	o	o

Display This Question:

If How many children aged 0-13 years do you currently provide care for? = 4

Q5.16 In your opinion, **how many minutes per day** did your children spend engaged in physical activity during Ontario's **strictest COVID-19 related lockdowns** (when sport and neighbourhood

closures were in effect; March 2020- June 2020; January 2021 - May 2021)? **Please respond as you have in previous questions of the survey, from YOUNGEST to OLDEST.**

	Less than 30 minutes per day (1)	30-59 minutes per day (2)	60-149 minutes per day (4)	More than 150 minutes per day (5)
Child 1 (1)	○	○	○	○
Child 2 (2)	○	○	○	○
Child 3 (3)	○	○	○	○
Child 4 (4)	○	○	○	○

Display This Question:

If How many children aged 0-13 years do you currently provide care for? = 4

Q5.17 In your opinion, how many **minutes per day** are your children engaging in physical activity **currently (i.e., at this moment in time)? Please respond as you have in previous questions of the survey, from YOUNGEST to OLDEST.**

	Less than 30 minutes per day (1)	30-59 minutes per day (2)	60-149 minutes per day (4)	150 minutes or more per day (5)
Child 1 (1)	○	○	○	○
Child 2 (2)	○	○	○	○
Child 3 (3)	○	○	○	○
Child 4 (4)	○	○	○	○

Display This Question:

If How many children aged 0-13 years do you currently provide care for? = 5

Q5.18 In your opinion, **how many minutes per day** did your children spend engaged in physical activity during Ontario's **strictest COVID-19 related lockdowns** (when sport and neighbourhood closures were in effect; March 2020- June 2020; January 2021 - May 2021)? **Please respond as you have in previous questions of the survey, from YOUNGEST to OLDEST.**

	Less than 30 minutes per day (1)	30-59 minutes per day (2)	60-149 minutes per day (4)	150 minutes or more per day (5)
Child 1 (1)	○	○	○	○
Child 2 (2)	○	○	○	○
Child 3 (3)	○	○	○	○
Child 4 (4)	○	○	○	○
Child 5 (5)	○	○	○	○

Display This Question:

If How many children aged 0-13 years do you currently provide care for? = 5

Q5.19 In your opinion, how many **minutes per day** are your children engaging in physical activity **currently (i.e., at this moment in time)? Please respond as you have in previous questions of the survey, from YOUNGEST to OLDEST.**

	Less than 30 minutes per day (1)	30-59 minutes per day (2)	60-149 minutes per day (4)	150 minutes or more per day (5)
Child 1 (1)	○	○	○	○
Child 2 (2)	○	○	○	○
Child 3 (3)	○	○	○	○
Child 4 (4)	○	○	○	○
Child 5 (5)	○	○	○	○

Display This Question:

If How many children aged 0-13 years do you currently provide care for? = 6

Q5.20 In your opinion, **how many minutes per day** did your children spend engaged in physical activity during Ontario's **strictest COVID-19 related lockdowns** (when sport and neighbourhood

closures were in effect; March 2020- June 2020; January 2021 - May 2021)? **Please respond as you have in previous questions of the survey, from YOUNGEST to OLDEST.**

	Less than 30 minutes per day (1)	30-59 minutes per day (2)	60-149 minutes per day (4)	150 minutes or more per day (5)
Child 1 (1)	○	○	○	○
Child 2 (2)	○	○	○	○
Child 3 (3)	○	○	○	○
Child 4 (4)	○	○	○	○
Child 5 (5)	○	○	○	○
Child 6 (6)	○	○	○	○

Display This Question:

If How many children aged 0-13 years do you currently provide care for? = 6

Q5.21 In your opinion, how many **minutes per day** are your children engaging in physical activity **currently (i.e., at this moment in time)? Please respond as you have in previous questions of the survey, from YOUNGEST to OLDEST.**

	Less than 30 minutes per day (1)	30-59 minutes per day (2)	60-149 minutes per day (4)	150 minutes or more per day (5)
Child 1 (1)	○	○	○	○
Child 2 (2)	○	○	○	○
Child 3 (3)	○	○	○	○
Child 4 (4)	○	○	○	○
Child 5 (5)	○	○	○	○
Child 6 (6)	○	○	○	○

Display This Question:

If How many children aged 0-13 years do you currently provide care for? = 7

Q5.22 In your opinion, **how many minutes per day** did your children spend engaged in physical activity during Ontario's **strictest COVID-19 related lockdowns** (when sport and neighbourhood

closures were in effect; March 2020- June 2020; January 2021 - May 2021)? **Please respond as you have in previous questions of the survey, from YOUNGEST to OLDEST.**

	Less than 30 minutes per day (1)	30-59 minutes per day (2)	60-149 minutes per day (4)	150 minutes or more per day (5)
Child 1 (1)	o	o	o	o
Child 2 (2)	o	o	o	o
Child 3 (3)	o	o	o	o
Child 4 (4)	o	o	o	o
Child 5 (5)	o	o	o	o
Child 6 (6)	o	o	o	o
Child 7 (7)	o	o	o	o

Display This Question:

If How many children aged 0-13 years do you currently provide care for? = 7

Q5.23 In your opinion, how many **minutes per day** are your children engaging in physical activity **currently (i.e., at this moment in time)? Please respond as you have in previous questions of the survey, from YOUNGEST to OLDEST.**

	Less than 30 minutes per day (1)	30-59 minutes per day (2)	60-149 minutes per day (4)	150 minutes or more per day (5)
Child 1 (1)	◯	◯	◯	◯
Child 2 (5)	◯	◯	◯	◯
Child 3 (6)	◯	◯	◯	◯
Child 4 (7)	◯	◯	◯	◯
Child 5 (8)	◯	◯	◯	◯
Child 6 (9)	◯	◯	◯	◯
Child 7 (10)	◯	◯	◯	◯

Display This Question:

If How many children aged 0-13 years do you currently provide care for? = 8

Q5.24 In your opinion, **how many minutes per day** did your children spend engaged in physical activity during Ontario's **strictest COVID-19 related lockdowns** (when sport and neighbourhood

closures were in effect; March 2020- June 2020; January 2021 - May 2021)? **Please respond as you have in previous questions of the survey, from YOUNGEST to OLDEST.**

	Less than 30 minutes per day (1)	30-59 minutes per day (2)	60-149 minutes per day (4)	150 minutes or more per day (5)
Child 1 (1)	○	○	○	○
Child 2 (2)	○	○	○	○
Child 3 (3)	○	○	○	○
Child 4 (4)	○	○	○	○
Child 5 (5)	○	○	○	○
Child 6 (6)	○	○	○	○
Child 7 (7)	○	○	○	○
Child 8 (8)	○	○	○	○

Display This Question:

If How many children aged 0-13 years do you currently provide care for? = 8

Q5.25 In your opinion, how many **minutes per day** are your children engaging in physical activity **currently (i.e., at this moment in time)? Please respond as you have in previous questions of the survey, from YOUNGEST to OLDEST.**

	Less than 30 minutes per day (1)	30-59 minutes per day (2)	60-149 minutes per day (4)	150 minutes or more per day (5)
Child 1 (1)	○	○	○	○
Child 2 (2)	○	○	○	○
Child 3 (3)	○	○	○	○
Child 4 (4)	○	○	○	○
Child 5 (5)	○	○	○	○
Child 6 (6)	○	○	○	○
Child 7 (7)	○	○	○	○
Child 8 (8)	○	○	○	○
